# Aquaculture rearing systems induce no legacy effects in Atlantic cod larvae or their rearing water bacterial communities

**DOI:** 10.1038/s41598-022-24149-x

**Published:** 2022-11-17

**Authors:** Madeleine S. Gundersen, Olav Vadstein, Peter De Schryver, Kari Johanne Kihle Attramadal

**Affiliations:** 1 Department of Biotechnology and Food Science, NTNU-Norwegian University of Science and Technology, Trondheim, Norway; 2 Laboratory of Aquaculture and Artemia Reference Center, Ghent University, Ghent, Belgium

**Keywords:** Microbial ecology, Environmental biotechnology

## Abstract

The microbial rearing quality influences the survival of marine larvae. Microbially matured water treatment systems (MMS) provide a more favourable rearing water microbiome than flow-through systems (FTS). It has previously been hypothesised, but not investigated, that initial rearing in MMS leaves a protective legacy effect in Atlantic cod larvae (*Gadus morhua*). We tested this hypothesis through a crossover 2 × 2 factorial experiment varying the rearing water treatment system (MMS vs FTS) and the microbial carrying capacity (+ /− added organic matter). At 9 days post-hatching, we switched the rearing water treatment system. By comparing switched and unswitched rearing tanks, we evaluated if legacy effects had been established in the larvae or their surrounding rearing water bacterial community. We analysed the bacterial communities with flow cytometry and 16S rRNA gene sequencing. We found no evidence that the initial rearing condition left a legacy effect in the communities by evaluating the bacterial community diversity and structure. Instead, the present rearing condition was the most important driver for differences in the rearing water microbiota. Furthermore, we found that MMS with high microbial carrying capacity appeared to seed a stable bacterial community to the rearing tanks. This finding highlights the importance of keeping a similar carrying capacity between the inlet and rearing water. Moreover, we reject the hypothesis that the initial rearing condition leaves a protective legacy effect in larvae, as the larval survival and robustness were linked to the present rearing condition. In conclusion, our results highlight the importance of maintaining a beneficial microbial rearing environment from hatching and throughout the larval rearing period.

## Introduction

Early-stage marine larvae have high mortality and are vulnerable to poor microbial rearing conditions, potentially resulting in infections and gut-dysbiosis^1^. However, beneficial fish-microbe interactions can increase survivability, growth, and resistance to detrimental bacterial colonization^2^. In land-based aquaculture, the fish and its microbiota are influenced by the rearing system conditions^3,4^, which can be controlled and managed to optimise fish growth and health^5,6^. Fish are in close contact with their surrounding water^7^, and it is now well established that the fish microbiota is influenced by, and changes with, its surrounding water microbiota^3^. The fish microbiome is shaped by many variables, including internal factors such as species, genetics and developmental stage^1^, and external factors such as feed, rearing system operation and environmental carrying capacity^8^. For this reason, efforts to manage the fish microbiota, and thereby minimize the impact of harmful microbial interactions, are important to increase the production in marine aquaculture.

The rearing water treatment systems can be operated to select for beneficial host microbes^3,9,10^. Disinfection of the intake water is an essential first line of defence against pathogenetic diseases^3^. However, disinfection reduces the bacterial biomass well below the carrying capacity of the system. This reduction results in an environment favouring the growth of opportunistic, often pathogenic, bacteria that thrive when resources are in surplus^3^. Conventional flow-through aquaculture systems (FTS) typically create environments favouring opportunists^9,11–13^. In FTS, the microbial carrying capacity of the rearing water is considerably higher than in the intake water^9^. This elevated microbial carrying capacity in the rearing tanks is due to an increased organic load from fish feed and faeces and a high hydraulic retention time (HRT) in tanks during larval rearing. Due to the low bacterial load after disinfection and the high microbial carrying capacity, rapid bacterial regrowth is observed in these environments, which are characterized as unstable, with low bacterial community diversity, a high fraction of opportunists and low biological control^3^.

However, by applying ecological theory to manage the microbiota of the rearing tanks, it is possible to select against the opportunistic bacteria^7^. Skjermo et al. 1997 proposed to mature the intake microbial community in a maturing biofilter unit to avoid the rapid regrowth in the rearing tanks^14^. In a maturing biofilter, the bacterial regrowth to the microbial carrying capacity of the intake water occurs under strong competition before entering the rearing tanks^14^. The maturing biofilter is inhabited by bacteria that compete for the incoming resources and therefore develops into a stable community dominated by competition specialists with a reduced risk of opportunistic proliferation. Stable competitive environments are characterised by higher diversity and the potential for higher biological control^3^. Using microbially matured systems (MMS) compared to FTS systems has resulted in increased larval viability of Atlantic halibut (*Hippoglossus hippoglossus*)^14^, turbot (*Scophthalmus maximus*)^14^ and Atlantic cod (*Gadus morhua*)^9^.

Microbial communities are assembled through deterministic and stochastic processes^15^, as we previously have shown for the microbiota of Atlantic cod rearing water^16^. Processes that happened in the past can leave deterministic legacy effects in the microbial community^17^ and the fish^18,19^. Attramadal et al. (2014) observed higher microbial community stability and Atlantic cod larval survivability in MMS compared to FTS systems^9^. The authors proposed that the increased survivability in MMS was due to a beneficial microbiome initially colonizing the larvae or the rearing water during the first days of rearing^9^. It was further claimed that this legacy effect should persist during larval rearing. However, that experiment was not designed to investigate legacy effects, and thus it has not been tested whether the initial rearing conditions leave a legacy effect.

This study investigated whether the initial rearing condition established legacy effects in Atlantic cod larvae or their rearing water microbiota. We used a 2 × 2 factorial crossover design with rearing water treatment systems (FTS vs MMS) and microbial carrying capacity (added extra organic matter or not) as the experimental variables. After nine days post-hatching, we switched the inlet water treatment system in half of the rearing tanks. We investigated whether the initial water treatment system left legacy effects in two of the system's biological components: the rearing water bacterial communities and the larvae. By comparing the bacterial communities in the rearing water in switched and unswitched tanks, we investigate if a legacy effect was established in terms of the diversity within each rearing tank (α-diversity) and the structure and taxonomic composition of the communities (β-diversity). We hypothesised that the MMS systems would have higher microbial stability and lower fractions of opportunistic and possibly detrimental species than the FTS systems. We increased the microbial carrying capacity in half of the tanks to evaluate the combined effect of treatment and increased population size of bacteria on the larvae. Similarly, we assessed if legacy effects were established in the larvae by determining if there were differences in the larval weight, robustness, and survival between switched and unswitched rearing treatments. Based on a previous study^9^, we hypothesised that the initial larval colonisation in MMS would leave a protective legacy effect in the larvae, resulting in increased survival and stress tolerance compared to the larvae reared in the FTS.

## Materials and methods

### Experimental design and setup

The experiment had a 2 × 2 factorial design with the rearing water system and microbial carrying capacity as the two factors and was operated for 20 days post-hatching (DPH). Halfway through the experiment (9 DPH), the inlet water treatment system was switched for half of the rearing tanks by changing the inlet water pipes. Intake water (70 m depth, Trondheimsfjord) was sand-filtered (50 µm) and UV-treated. Half of the 16 rearing tanks (100 L, black, coned bottom) received this water directly and were operated as FTS. For the remaining eight tanks, the intake water was microbially matured in a biofilter (MMS) before entering the rearing tanks^12^. The microbial carrying capacity was manipulated by adding 20 mg/L of organic matter daily directly to each FTS rearing tank (FTS+) and the biofilter serving the MMS rearing tanks (MMS+). The organic matter was a mix of tryptone, peptone and yeast extract (6.67 mg/L each). The tanks with added organic matter were characterized as having a high microbial carrying capacity (+), whereas the others had a low capacity (−). We refer to the rearing tanks that switched water treatment during the experiment as, for example, ‘MMS+ to FTS+’ to indicate that the tanks received MMS+ water for the first nine DPH before switching to the FTS+ treatment for the rest of the experiment. The carrying capacity was not changed for any of the tanks throughout the experiment.

### Rearing regime and biofilter pre-cultivation

The Atlantic cod were reared for 20 DPH. Atlantic cod eggs (Havlandet Marine Yngel AS) were disinfected with glutaraldehyde for 10 min (400 ppm) and rinsed in disinfected seawater for 30 s^20^. The larvae hatched at 90–95-day degrees (°d). The experiment was conducted within the Norwegian animal welfare act guidelines^21^. The Norwegian Animal Research Authority (NARA) approved the facility and this experiment under id 6729. This study is reported according to the ARRIVE guidelines (https://arriveguidelines.org/).

Each experimental tank was stocked with larvae (100 larvae/L) and maintained in darkness until 3 DPH, after which they were kept in continuous light. The tank water exchange rate started at 2 and increased to 4 tank volumes day^−1^ at 8 DPH. A feeding robot (Storvik, Norway) added suspended clay (Vingerling K148, WBB Fucs GmbH, Germany) to the fish tanks (0.1 g L^−1^ day^−1^) from 1 DPH^22^. Larvae were fed rotifers from 3 DPH and a mix of rotifers and artemia from 18 DPH (Supplementary Table 1).

The two biofilters (267 L) were filled 25% with used Kaldnes carriers K1 (Anox Kaldnes) from the same source biofilter. The biofilters were pre-cultivated to ensure that the biofilm had formed sufficiently and that the microbial communities had stabilised. Six weeks before hatching the two biofilters were operated as batch at 20 °C and fed every second day with 20 mg/L of the organic matter mix. Four weeks before hatching, water and carriers from the two biofilters were mixed to ensure similar biofilm composition. At the same time, the temperature was lowered to 13 °C, each system refilled with 50 L fresh water, and the flow rate increased to 10 L/h. Onwards, the fed-biofilter (MMS+) was added 20 mg/L of organic matter daily, while the MMS− only received incoming fjord water. Three weeks before hatching, the flow rate was increased to 20 L/h.

### Larval growth

The larval growth was quantified by weighing the freeze-dried larvae individually (9–10 larvae per tank at 4, 8 and 12 DPH) or as a pool (3–5 larvae and 5 samples per tank at 2, 12 and 17 DPH). Due to high mortality in the FTS+ tanks, data is lacking from rearing tanks connected to that system at 12, 17 and 18 DPH. Larvae were sacrificed with an overdose of MS222 and rinsed with dH_2_O.

### Larval stress tolerance

The robustness of the larvae was evaluated as percent survival after exposure to different stress tests on 8, 11 and 17 DPH in two side experiments. The two experiments tested the general stress level of the larvae through a “transfer challenge” and the larvae’s resistance to invasion stress through a rearing water “invasion challenge”. The transfer challenge can be interpreted as a negative control to the invasion challenge as it only reflects the stress of being transferred from the main rearing tank.

Larvae were harvested by siphoning with silicone hose throughout the tank from one or both rearing tanks in each rearing treatment. An exception was tanks with FTS+ as the initial rearing treatment due to high mortality (see Supplementary Table 2 for subsampling overview). The transfer challenge reflecting the general stress of the larval was conducted on 11 and 17 DPH by simply transferring 10–12 larvae and 100 mL of rearing water from each tank to sterile Nunc culture flasks). The invasion challenge was performed on 8, 11 and 17 DPH. First, we transferred 3.5 L of rearing water to a glass bowl and invaded it with 1.5–2.8 × 10^6^ *Pseudoalteromonas* CFUs/mL and 2.8 × 10^4^ *Polaribacter* CFUs/mL in a glass bowl (see details below). Next, 2 × 100 mL of this invaded rearing water was transferred to two sterile Nunc culture flasks and 10–12 larvae were added to the flasks. Thus, we had n = 1 and n = 2 flasks per subsampled tank for the transfer and invasion challenge, respectively. After 24 h, the survival of the larvae in the flasks was determined.

The two bacteria used as the invaders had previously been isolated from the system on marine agar plates and preserved in 20% glycerol at −80 °C. The bacterial DNA was extracted using ZymoBIOMICS MagBead DNA/RNA extraction kit before the 16S rRNA gene was amplified using the broad coverage PCR primers Eub8F (5′-AGAGTTTGATCMTGGCTCAG-3′) and 1492R (5′-GGTTACCTTGTTACGACTT-3′). The reactions were run for 38 cycles (98 °C 15 s, 55 °C 20 s, 72 °C 20 s) with 0.3 μM of each primer, 0.25 mM of each dNTP, 1× Phusion buffer HF, 0.015 units/μL of Phusion Hot StartII DNA polymerase, 1 μL of DNA template and dH20 to a total volume of 25 μL. The DNA sequences were obtained through Sanger sequencing (LightRun, Eurofins). The bacteria were identified to belong to the *Pseudoalteromonas* and the *Polaribacter* genera, respectively, through the SeqMatch function of the RDP database^23^.

### Survival in rearing tanks

Survival was quantified at 20 DPH as the percentage of remaining larvae compared to initial stocking in each tank. The remaining larvae at 20 DPH were sacrificed as described above before counting.

### Bacterial density and net growth potential

The bacterial density in incoming- and rearing water was quantified using flow cytometry (BD accuri C6) in samples collected at 2, 9, 11 and 15 DPH. Each sample was split into two aliquots; one was fixated immediately with 1% glutaraldehyde and used to quantify the bacterial density. The other was incubated as is in the fish rearing room in cell culture tubes without shaking for three days before fixation. The incubated samples were used to determine the net growth potential of the bacterial community. We calculated the growth potential as the logarithmic (base 2) ratio between the bacterial density in incubated and non-incubated samples. Thus, the net growth potential represents the number of doublings in density after incubation. We defined samples as being at the microbial carrying capacity if the net growth potential was < 0.

### 16S rRNA gene amplicon library preparation and sequencing

The bacterial communities of the rearing water were filtered through Dynaguard syringe filters (0.2 µm, 50 mL) at 1 and 12 DPH and stored at −80 °C until DNA extraction. DNA extraction and amplicon library preparation was carried out as described in Gundersen et al. 2021^24^. Briefly, bacterial community DNA was extracted using the Qiagen DNeasy PowerSoil DNA extraction kit. Then, broad-coverage primers were used to amplify the V3-V4 region of the 16S rRNA gene using PCR. The amplicon library was then normalised and purified before amplicon indexing with the Illumina Nextera XT Index kits (FC-131-2004). Finally, the amplicon library was sequenced with Illumina MiSeq at the Norwegian Sequencing centre^25^. The sequencing reads are deposited at the European Nucleotide Archive (accession number ERR9837055-ERR9837086). The 16S rRNA gene amplicon dataset contained 450,369 sequence reads with a mean sequencing depth of 14,074 (± 6418 SD) reads per sample.

### Processing of Illumina sequence data

The USEARCH pipeline was used to process the Illumina sequence data^26^. First, paired ends were merged simultaneously as primer sequences and reads shorter than 400 bp were removed. Then Unoise3 was used to perform error correction of the amplicon reads, and an amplicon sequence variant (ASV) table was generated^27^. Finally, ASV sequences were taxonomically assigned to the rdp dataset (rdp16s_v18)^23^ version 18 at an 80% confidence level using the sintax command^28^ yielding 1315 ASVs.

All data analysis was subsequently performed in R version 4.1.0^29^. First, ASV sequences were multi-aligned using the *AlignSeqs*() function from the DECIPHER package^30^. Then the phangorn package was used to construct a phylogenetic tree from the alignment using neighbour-joining, which was fitted to a generalised time-reversible maximum likelihood tree^31^. All ASVs with less than 8 reads and those identified as non-bacterial were removed from the dataset. Next, the tree was rooted to the longest branch using *root*() from the package ape^32^. Next, each sample was scaled to the lowest sequence depth using *transform*() from the package microbiome^33^. This scaled dataset was rarefied using *rarefy_even_depth*() from phyloseq to ensure equal sampling depth^34^. An assessment of the 16S rRNA gene amplicon dataset quality can be found in Supplementary materials online. All plots were generated using the packages ggplot2^35^ and ggpubr^36^.

### Statistical analysis

The α-diversity was estimated as Hill diversity of order 0 (i.e. richness) and 1 (i.e. exponential Shannon)^37^**.** These diversity numbers were estimated using the function *reyni*() from vegan^38^. One-way analysis of variance (ANOVA) was used to test for differences in α and β-diversity and larval weight means between groups^39^. The data were tested for homoscedastic variance using the Flinger-Killeen test^40^ and for normal distribution with the Shapiro–Wilk’s test^41^ using the functions *fligner.test*() and *shapiro.test(),* respectively. When the requirements for ANOVA were not met, the Kruskal–Wallis test was used^42^. The Tukey test^43^ was used for post hoc comparisons of group means using the function *TukeyHSD().*

To investigate differences in community composition between samples, we calculated the Bray–Curtis and UniFrac distance and their incidence-based equivalents Sørensen and unweighted UniFrac distance. The distance matrixes were calculated with *distance*(), ordinated with a principal coordinate analysis (PCoA) using *ordinate*() and plotted using *plot_ordination*() from phyloseq^34^.

We used DeSeq2 to perform a differential abundance test. DeSeq2 quantifies which ASVs that have significantly different abundances between groups^44^. Briefly, the un-normalized ASV table was used for the DeSeq2 analysis. First, the count data were median ratio normalised using *etimateSizeFactors*(). Then, the dispersion for each ASV was estimated using *estimateDispersion*(). A Wald significance test was then performed on a parametric fitted negative binomial GLM model using *DESeq*(test = ” Wald”, fitType = “parametric”).

## Results

### Bacterial density and growth potential in the rearing water were related to the microbial carrying capacity

**Figure 1:**
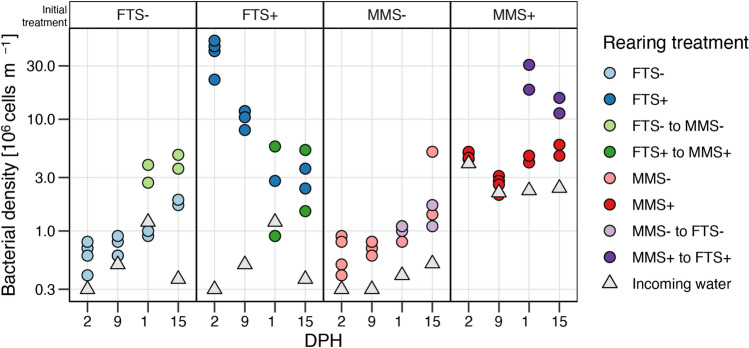
Bacterial density (million bacterial cells mL^−1^) at various days post-hatching (DPH) in incoming and rearing tank water. Note that the y-axis is log scaled. Colours indicate the rearing treatment, and shape signifies rearing (filled circle) and incoming water (filled triangle).

Quantifying the bacterial density in each tank verified that we obtained a higher bacterial load in the systems with added organic material. The bacterial density was, on average, 7.8× higher in the systems with high compared to low bacterial carrying capacity. This difference was particularly evident at 2 (34.8×, Kruskal–Wallis p = 0.0008) and 9 DPH (9.1×, Kruskal–Wallis p = 0.0007) (Fig. 1). The bacterial density increased throughout the experiment for the tanks with low microbial carrying capacity (treatment group MMS−, FTS−), reflecting increased larval feeding and defecation. Contrastingly, the bacterial density was relatively stable over time in the MMS+ treatment and even decreased over time in the FTS+ treatment. When averaging the densities at 11 and 15 DPH within each rearing treatment, we observed that the ‘MMS+ to FTS+’ had a considerable difference in the bacterial density between incoming and rearing water (24.2×). In contrast, this difference was below 8.2× in all other treatment tanks. Such differences in density indicated that some communities were below the microbial carrying capacity of the systems. We thus investigated the growth potential to determine if carrying capacity was reached in the rearing water.

The bacterial net growth potential in the intake and rearing water was quantified as the number of cell doublings after incubation for 3 days^11^. Generally, the FTS− and MMS− rearing water had net growth potential with an average of 0.2 and 0.1, respectively (Supplementary Fig. 2). In contrast, the rearing water of the FTS+ and MMS+ had a negative net growth potential with averages of −0.2 and −0.06, respectively. In the case of negative net growth potential, the bacterial density decreased during the incubation. A negative net growth potential suggested that the rearing water bacterial communities were at the tank’s microbial carrying capacity at the time of sampling. Thus, the bacterial communities were at the carrying capacity of the high (+) carrying capacity systems and below in the low (−) systems. To gain a deeper understanding of the bacterial community characteristics the 16S rRNA gene of the bacterial community was sequenced at 1 and 9 DPH.

### Initial rearing condition did not leave a legacy effect on bacterial α-diversity

**Figure 2:**
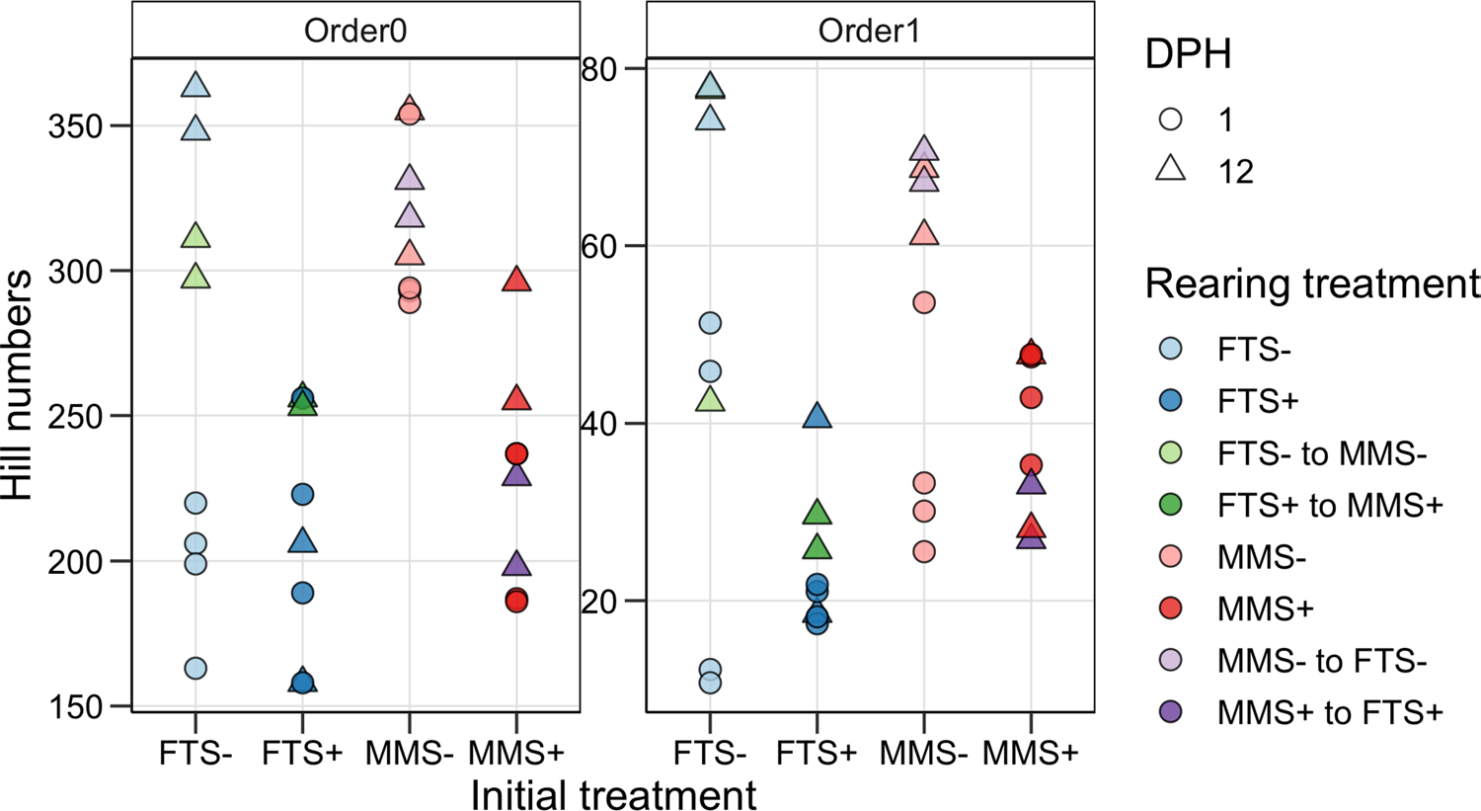
The bacterial α-diversity of Hill diversity orders 0 and 1 at 1- and 12-days post-hatching (DPH). Colours indicate rearing treatment, and shape signifies 1 (filled circle) and 12 DPH (filled triangle). Hill diversity of order 0 is equivalent to ASV richness, and order 1 is equal to exponential Shannon, which also accounts for ASV abundances.

The bacterial α-diversity of the rearing water was investigated at 1 and 12 DPH (Fig. 2). At 1 DPH, the richness was comparable between the FTS−, FTS+ and MMS+ treatments, but on average, 1.5× higher for the MMS− treatment (307 vs 205 ASVs, Tukey’s test p < 0.006). The diversity of order 1 was, on average, 1.5× higher for the MMS+ and MMS− treatments than for the FTS+ and FTS− treatments (ANOVA p = 0.05).

We were interested in determining whether the initial rearing system had a legacy effect on α-diversity. We first evaluated whether there were differences between the unswitched treatments at 12 DPH. For the high carrying capacity treatments, the MMS+ had, on average more ASVs than the FTS+ treatment (275 vs 182 ASVs, Tukey’s test p = 0.04). For the low carrying capacity group, the MMS− group had, on average fewer ASVs than the FTS- treatment (330 vs 356 ASVs, Tukey’s test p = 0.9). Note that statistical tests with data from 12 DPH have low power (n = 2 replicates/group). Comparing the switched tanks to those that continued with the initial treatment showed that ‘FTS− to MMS−’, ‘FTS+ to MMS+’ and ‘MMS+ to FTS+’ had a more similar richness to their post-switch treatments. Only the ‘MMS− to FTS−’ treatment had a more similar richness to the initial treatment. However, only 25 ASVs, on average, differentiated MMS− and FTS−. We thus conclude that the initial rearing treatment did not leave a legacy effect on richness. Similarly, there was no indication that the initial rearing treatment had a legacy effect on the diversity of order 1.

However, we did observe that the richness had increased in all treatments, except in the tanks continuing with FTS+. The increase in richness was similar in the tanks with low carrying capacity (FTS− and MMS−) regardless of whether the tank changed water treatment system or not. However, for the tanks with FTS+ as the initial treatment, the richness decreased 0.88× in the tanks continuing with FTS+ but increased 1.2× for tanks that switched to the MMS+ system. Interestingly, the opposite was observed for the tanks starting with MMS+. For these, the richness increased 1.3× in the tanks continuing with MMS+ but was stable for those that switched to FTS+ (1.0×). There were few differences in diversity of order 1 between the switched and unswitched treatments at 12 DPH. However, the diversity of order 1 had increased in all treatments, except in the tanks starting with the MMS+ treatment.

We interpret the increases in α-diversity as indicating that the bacterial communities were unstable at 1 DPH, thus allowing the inlet bacteria to disperse and establish. Notably, the decrease in diversity of order 1 in the tanks starting with MMS+ suggests that these bacterial communities were stable, more even, and resisted the establishment of the microbiota from the new intake water source (e.g. ‘MMS+ to FTS+’ had stable richness, and decreased 0.7× in diversity of order 1). The stability of the MMS+ bacterial communities was also supported by the β-diversity.

### The MMS+ rearing bacterial community was most stable over time

**Figure 3:**
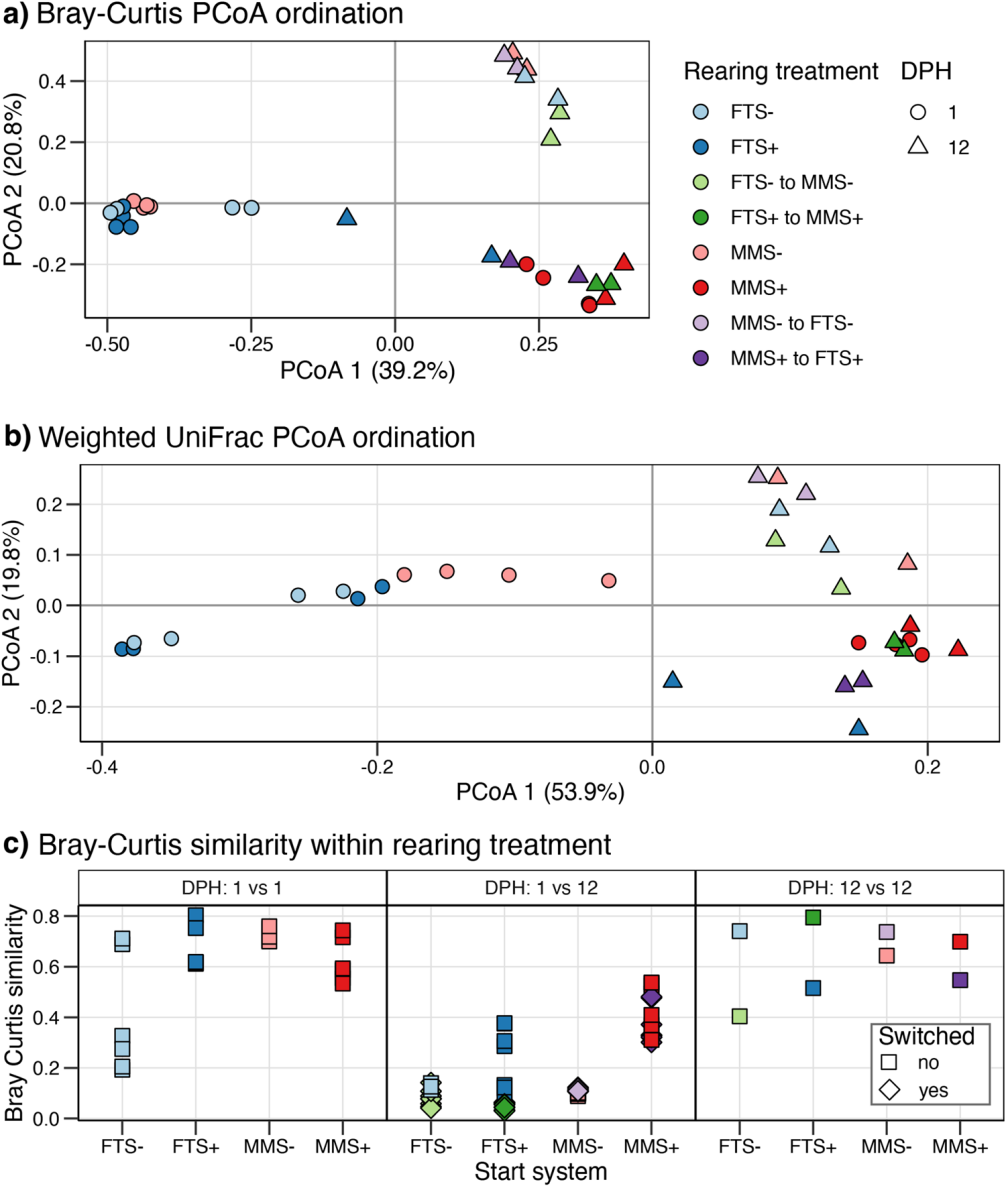
Community composition comparisons between samples (β-diversity) based on rearing treatment. PCoA ordinations are based on (**a**) Bray–Curtis or (**b**) weighted UniFrac distance. Colours indicate rearing treatment, and shape signifies 1 (filled circle) and 12 DPH (filled triangle). (**c**) The Bray–Curtis similarity within rearing treatment within and between sampling days. Colours indicate rearing condition and shape unswitched (filled square) and switched (filled diamond) treatments.

The differences in bacterial community composition between samples were quantified using Bray–Curtis and the weighted UniFrac distances and then ordinated using PCoA (Fig. 3). The PCoA ordinations indicated that most of the differences in community composition were explained by sampling day and rearing treatment (Fig. 3a,b). The MMS+ samples clustered oppositely from the other three rearing treatments along Axis 1 at 1 DPH. Axis 1 explained 39.2% (Bray–Curtis) and 53.9% (UniFrac) of the variation in the distance matrixes, indicating that there was a large difference in community composition between MMS+ and the other treatments. At 1 DPH, the FTS+, FTS− and MMS− clustered together in the Bray–Curtis ordination but were more spread out when using the weighted UniFrac distance. As UniFrac is based on phylogenetic community dissimilarity, this spreading indicates that the ASVs that contributed to community differences between treatments were more different phylogenetically.

At 12 DPH, the differences in the bacterial community composition were separated based on the microbial carrying capacity along Axis 2. This axis explained 20.8% (Bray–Curtis) and 19.8% (UniFrac) of the variation. Moreover, we observed that all 12 DPH samples clustered closer to the 1 DPH MMS+ samples regardless of rearing treatment. This pattern indicated that succession drove the communities toward a common bacterial community composition. The MMS+ samples had already obtained this composition at 1 DPH, highlighting the advantage of pre-feeding the biofilter to acquire a stable microbial community composition.

The stability of the bacterial community composition was investigated by quantifying the within-system Bray–Curtis similarity within and between sampling days (Fig. 3c). The tanks starting with the MMS+ treatment had the highest bacterial community similarity when comparing 1 and 12 DPH with an average Bray–Curtis similarity of 0.4. In comparison, the Bray Curtis similarity was, on average, 0.1 in tanks starting with the other treatments (Kruskal–Wallis p < 0.001).

Next, we evaluated if the initial rearing condition had left a legacy effect on community composition. We compared the Bray–Curtis similarity at 12 DPH between switched and unswitched communities. Unfortunately, we could not perform statistics on these observations due to low power within the groups. The ‘FTS− to MMS−’ bacterial communities had an average Bray–Curtis similarity (± SD) of 0.4 (± 0.08) and 0.6 (± 0.05) to the communities of the MMS− and FTS−, respectively. The ‘MMS− to FTS−’ samples showed a similar pattern, with slightly higher similarity to communities continuing with the same initial treatment with average Bray–Curtis similarities of 0.6 (± 0.03) and 0.5 (± 0.01) to the MMS- and FTS- treatments, respectively. Thus, some legacy effects on the bacterial community composition might have established in both the MMS- and the FTS- tanks, but these effects were minor. Clearer patterns were observed in the conditions with high carrying capacity.

The bacterial communities switching from ‘MMS+ to FTS+’ resisted a change toward the FTS+ community structure. Instead, these ‘MMS+ to FTS+’ communities had higher Bray–Curtis similarities to the communities continuing with the MMS+ treatment (0.5 ± 0.1) than tanks that initially got the FTS+ treatment (0.2 ± 0.05). This is an indication of a legacy effect in the MMS+ rearing tanks. However, we observed the opposite for the ‘FTS+ to MMS+’ communities, which had higher Bray–Curtis similarity to the MMS+ communities (0.7 ± 0.06) than those continuing with FTS+ (0.4 ± 0.07). Thus, there was no legacy effect in the FTS+ rearing tanks. Due to the inconsistent patterns, we conclude that the initial rearing condition does not leave a legacy effect on the bacterial community composition. Instead, the mature biofilter (MMS+) supplied a bacterial community that was able to establish quickly in the tanks that previously were FTS+. To evaluate if the MMS+ biofilter seeded a bacterial community, we investigated the taxonomic composition of the samples.

### The bacterial community composition in the MMS+ rearing tanks differed taxonomically from those of the other treatments

**Figure 4:**
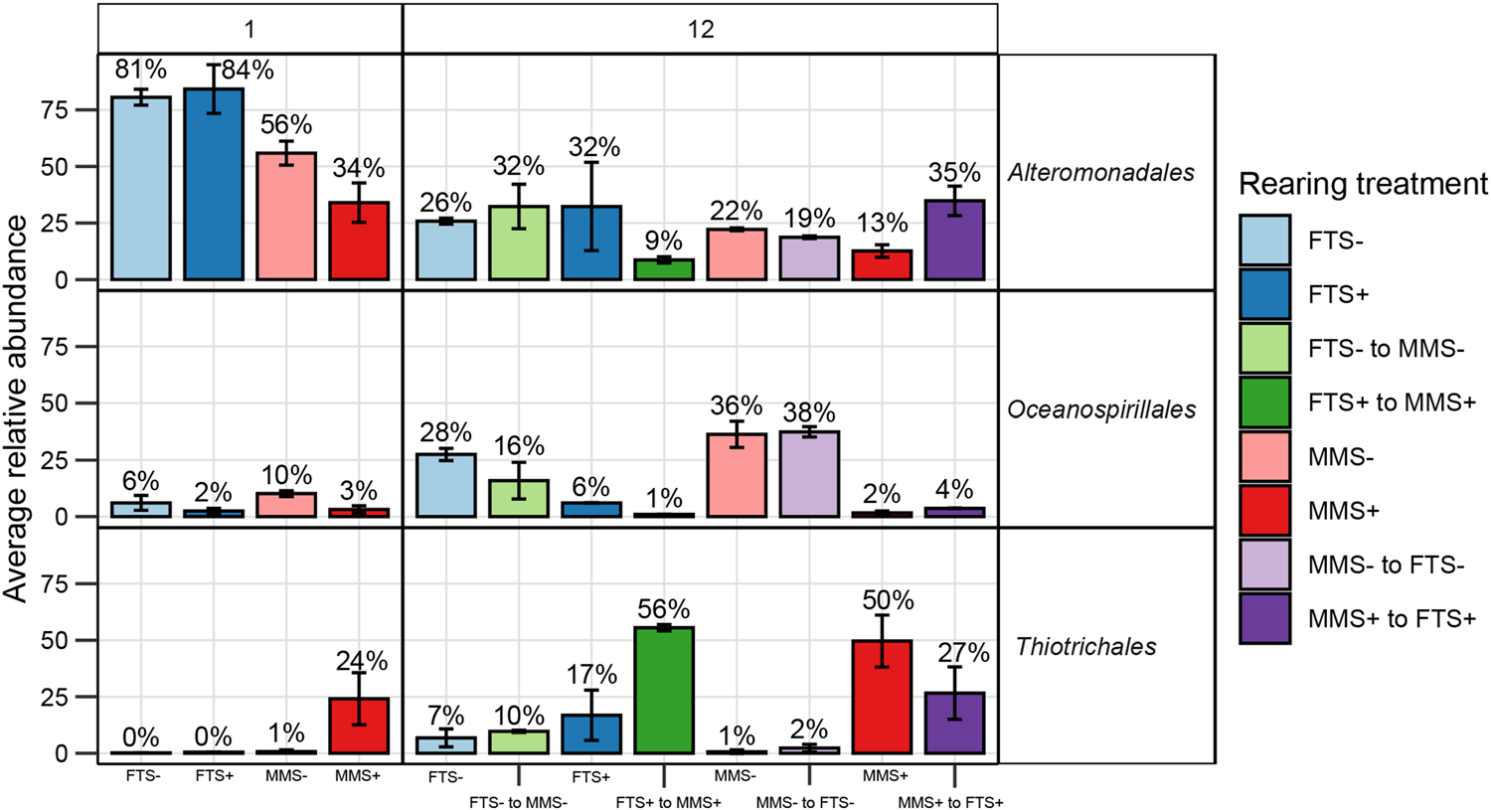
The relative abundance of the three most dominating orders in the dataset. These orders had a > 20% abundance in a minimum of two samples. Colours indicate the rearing treatment. The average relative abundance is shown on each sampling day, and whiskers represent the standard deviation.

The class *Gammaproteobacteria* dominated the rearing water in all treatments with an average relative abundance of 76 (± 11% SD). At the order level, we observed differences based on sampling day and rearing treatment (Fig. 4). At 1 DPH, the FTS−, FTS+ and MMS− were similar in bacterial composition, with a high abundance of *Alteromonadales*. The composition was different in the MMS+ rearing water, with substantially lower abundances of *Alteromonadales* and high abundances of *Thiotrichales*. At 12 DPH, the abundance of *Thiotrichales* had doubled in the MMS+ treatment from an average of 24% to 50%. Interestingly, this order also increased in the rearing tanks that switched from ‘FTS+ to MMS+’. Its abundance was 56% in the ‘FTS+ to MMS+’ tanks but only 17% in the FTS+ tanks. This noteworthy difference in abundance indicated that the biofilter community was effectively seeded to the rearing tanks. Next, we investigated if the rearing treatments affected larval viability.

### The present rearing treatment had the largest effect on larval performance

Comparing the larval dry weight between the treatments at each sampling day did not indicate that the rearing conditions affected the growth (Supplementary Fig. 2). At 17 and 18 DPH, there was no statistically significant difference between the average weight in the different rearing treatments. However, differences were observed in larval robustness.

**Figure 5:**
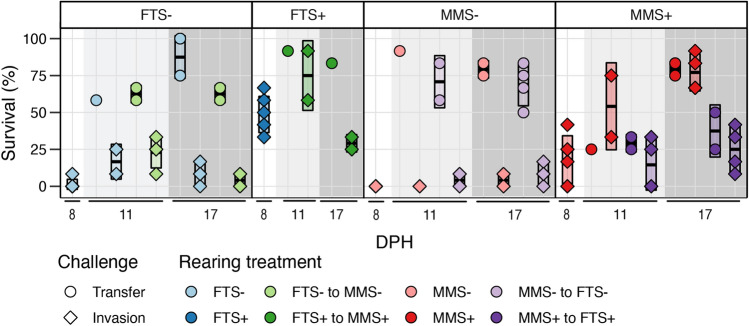
Percent of surviving larvae one day after the transfer and invasion challenge tests at various DPH. Samples are organized based on the initial rearing treatment. Colours indicate the overall rearing treatment. Boxplots represent mean survival ± SD for each rearing treatment at each sampling day.

The robustness of the larvae was investigated in side experiments on 8, 11 and 17 DPH by inducing stress through transfer or exposing the larvae to rearing water invaded with a *Pseudoalteromonas* and a *Polaribacter* bacterial strain (Fig. 5). While *Polaribacter* has been identified as a commensal^16,45^, *Pseudoalteronomonas* contains many pathogenic strains towards Atlantic cod^46^. The invaded rearing water thus pose a threat both through an increased bacterial load and exposure to a potentially pathogenic bacterium. Larval mortality was recorded 24 h after the challenge. Not surprisingly, the survival was higher for the larvae only challenged by transfer (mean 68.1 ± 21.2%) compared to larvae transferred to invaded rearing water (mean 20.5 ± 24.8%).

For the larvae that only were subjected to the transfer challenge, differences were observed between the rearing treatments. On average, the survival of larvae was comparable between the FTS−, FTS+ and MMS− treatments but was 1.5× lower for the MMS+ (Fig. 5). Generally, there was no indication that the initial rearing condition affected the general stress level of the larvae. Instead, robustness appeared to be related to the present rearing regime. For example, on 17 DPH, the larvae that continued with MMS+ had 2.1× higher survival than those that switched to FTS+ (i.e. ‘MMS+ to FTS+’). Thus, the initial rearing condition left no legacy effect on the general stress level of the fish.

For the invasion challenge, the larvae from tanks with low carrying capacity were the least robust. For these larvae, the mean (± SD) survival was 6.3 (± 8.6)%, and some flasks had 0% survival. In comparison, the larvae from tanks with high carrying capacity had a mean survival of 39.4 (± 26.8)% after invasion stress (Fig. 5). The data from the challenge tests did not indicate that the initial rearing condition left legacy effects on the larval robustness. For example, larvae from tanks that continued in MMS+ challenged with invasion had high survival [mean 69.4 (± 20.2)%], whereas larvae from the tanks that switched from ‘MMS+ to FTS+’ had 3.5× lower survival [mean 19.8 (± 16.0)%]. Unfortunately, we do not have samples from the FTS+ rearing treatment after 8 DPH due to high mortality in the rearing tanks. If there was a legacy effect, one would expect improved robustness to invasion when switching to a rearing regime associated with higher survival. Furthermore, the larval survival after the challenges was comparable between the FTS− and ‘FTS− to MMS−’and between the MMS− and ‘MMS− to FTS−’. In conclusion, there was no indication of a legacy effect in the larvae. Instead, the post-switch rearing treatment had the largest impact.

### Larval survival was very low in FTS+ tanks

**Figure 6:**
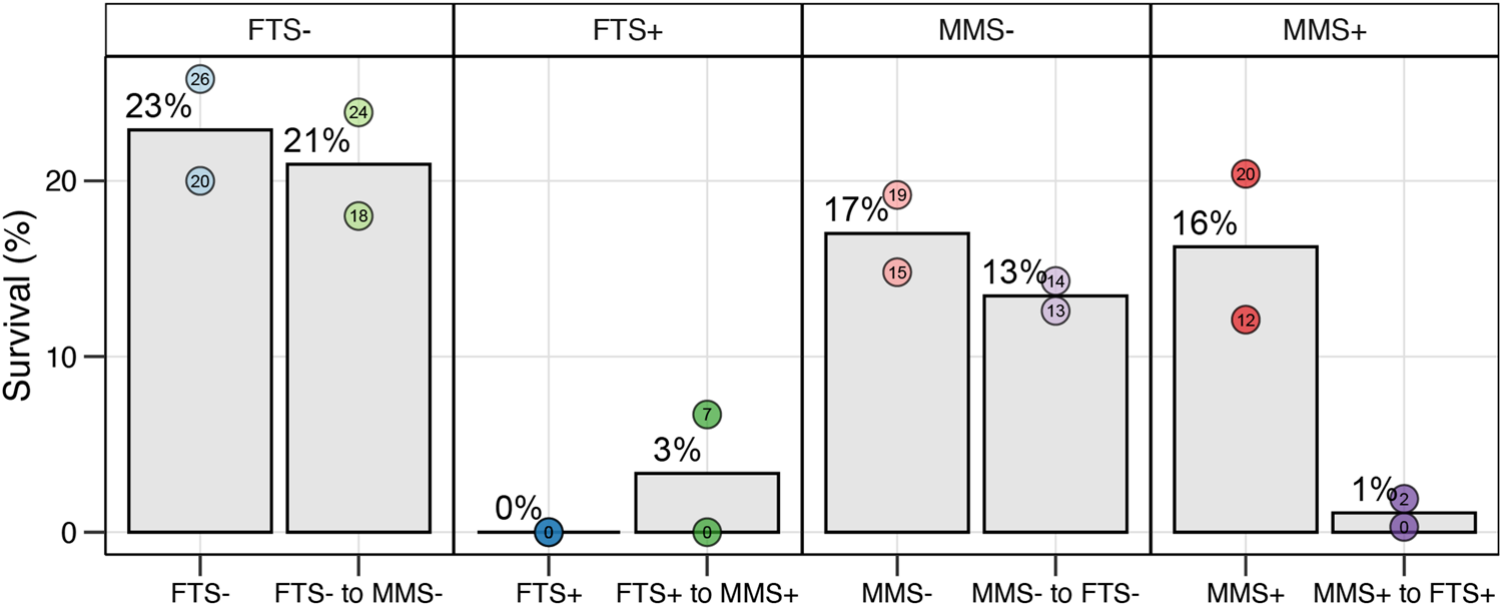
The survival in each rearing treatment at the end of the experiment at 20 DPH. The grey bars and percentages indicate the mean survival in the rearing tanks, whereas the points show each tank's survival.

Larval survival at the end of the experiment was comparable and relatively high for the MMS+ , MMS− and FTS− treatments. In these treatments, the survival ranged between 12 and 26%. However, survival was low for all tanks that at some point received FTS+ water, ranging from 0 to 7% (Fig. 6). It should be noted that the water quality was visually poorer in the FTS+ tank water. Nevertheless, we investigated if any ASVs were linked to survival.

We identified ASVs with significant log-fold changes between the bacterial communities in high and low survival tanks using a DeSeq2 analysis. Fifty-two ASVs had higher abundances in the communities from tanks with low survival, and 85 had higher abundances in those with high survival (FDR adjusted p-value < 0.05, Supplementary Fig. 3). An interesting pattern emerged when investigating the abundance of the identified ASVs in each rearing tank (Supplementary Fig. 4). At 1 DPH, the abundance of ASVs associated with low survival was over 40% in FTS+, FTS− and MMS− but below 20% in the MMS+ tanks.

When comparing switched and unswitched treatments at 12 DPH, it was apparent that the abundances of these low survival-associated ASVs were treatment dependent. For example, the abundances of these ASVs were 55% in the FTS+ treatment but 3.7× lower in the ‘FTS+ to MMS+’ treatment. The opposite was observed between MMS+ and ‘MMS+ to FTS+’. The low survival associated ASVs were only present at 1% in the MMS+ rearing tanks but increased to 15% in the ‘MMS+ to FTS+’ tanks. Furthermore, we found five ASVs classified as *Moritella* to be especially interesting. These five ASVs all had over a 7.5-log2 fold increase in the low survival tanks. Four of these ASVs were most similar to the type strain *Moritella viscosa*, a known fish pathogen (Supplementary Table 3, similarity > 92%). Our findings show that the rearing conditions can be used to select for a beneficial microbial environment for the larvae.

## Discussion

Marine larvae are hard to rear due to their vulnerability and poor viability associated with high mortality. However, microbial management of the rearing conditions, such as disinfecting the eggs^20^ or microbially maturing the water before it reaches the fish^14^, has resulted in considerable improvements in larval performance. These improvements indicate that microbial water quality is a major cause of poor larval viability.

We have previously hypothesised that the initial microbial rearing conditions leave legacy effects in Atlantic cod larvae^9^, but this had not been tested. Thus, in this paper, we investigated if legacy effects could be observed after initial rearing in either MMS or FTS water treatment in a unique and novel experimental design. This experiment’s unique feature was switching the incoming water treatment system in half of the rearing tanks at 9 DPH. This design had two major advantages compared to traditional designs. Firstly, only the inlet system pipes were changed at the water treatment switch. Thus, the larvae were never exposed to the stress of transfer between tanks, allowing us to measure the actual effect of the input microbiota. Secondly, as half of the tanks continued with the same rearing treatment, we could evaluate if legacy effects were established by investigating the larval performance and the microbial community characteristics. Overall, there was no evidence that the initial rearing conditions left a legacy effect in the larvae or their surrounding microbial communities after switching to novel conditions at 9 DPH.

Comparing switched and unswitched rearing regimes did not indicate a legacy effect in the bacterial community of the rearing water. Instead, we found that the microbial carrying capacity and the post-switch water treatment system were the main determinants for differences in bacterial density, growth potential, α-diversity and community composition. However, we observed that the bacterial community of the rearing water that switched from ‘MMS+ to FTS+’ had higher Bray–Curtis similarity to the community of the rearing water in MMS+ than FTS+ water. This observation could indicate a legacy effect in the MMS+ tanks. However, given that no other analysis pointed to a legacy effect, it instead seems like these observations reflect the stability of the MMS+ biofilter biofilm and the dominant effect of this biofilm on the rearing water. It is likely that this biofilm supplied, or seeded, a stable flow of inlet bacteria to the rearing water. This biofilm seeding effect was highest in the MMS+ treatment. The water provided by the MMS+ had a 10× higher bacterial density than the water from the MMS-. This difference in bacterial load might explain why the seeding effect was less pronounced in the MMS- rearing tanks. From these results, we recommend using microbially matured water at a carrying capacity similar to the carrying capacity in the rearing water to obtain a stable microbial rearing environment. This is in accordance with previous studies^7^.

Compared to the rearing water, the fish as a microbial ecosystem is less affected by the high water exchange rates. As such, we wanted to see if a legacy effect established in the larvae. Neither survival, weight, nor robustness of the larvae indicated that the larvae experienced a legacy effect based on the initial rearing conditions. Theoretically, for a legacy effect to establish, the effect needs to manifest in a deterministic way. Fish as an ecosystem is not stable at 20 DPH^47^. Due to large morphological and physiological changes during larval development, the niches available on and in the fish will change^48,49^. The lack of a legacy effect in Atlantic cod larvae is consistent with legacy-effects studies in other fish species, such as Nile tilapia (*Oreochromis niloticus*)^50^ and zebrafish (*Danio rerio*)^49^. This consistency poses the question of whether one can detect legacy effects in fish larvae that, through development, change the deterministic constraints of their environment. It has been documented that both deterministic and stochastic processes structure the fish larvae's microbiome ^16,51^. However, it is unclear what drives the deterministic processes, if these drivers are stable over time, and if the initial environmental conditions can impact them. In this experiment, we could not find that the initial rearing condition established legacy effects in the system. However, other drivers might be affected by historical effects. Our findings suggest that the fish developmental stage and environmental microbiota have most impact on the fish microbiota composition.

Instead of a legacy effect, the present rearing condition appeared important for larval survival and robustness. Especially pronounced was the low larval survival in tanks that at some timepoint were connected to the FTS+ rearing treatment (range 0–7%). In a previous experiment, the survival of Atlantic cod larvae was 65% higher in MMS compared to FTS systems at 32 DPH^9^. In that experiment, the rearing water system did not impact the bacterial community stability. The authors hypothesised that the higher survival in MMS was linked to a beneficial initial microbial colonisation and earlier onset of growth of the larvae upon mouth opening. Whilst we did observe a major difference in survival between larvae reared in MMS+ and FTS+, no difference was observed between MMS- and FTS-. Most importantly, there was no difference in survival between tanks reared entirely in FTS+ and those that switched from ‘MMS+ to FTS+’. Thus, the initial protective colonization from an assumed more beneficial microbiome (MMS) did not remain in the larvae.

This protective effect was also lacking in terms of larval robustness. Rather than a legacy effect, the largest differences in larval robustness were based on the microbial carrying capacity of the tanks. We observed that larvae from tanks with high carrying capacity were more robust to invasion by bacteria than those reared in low carrying capacity. This difference is likely related to the propagule pressure or the relative abundance of the invader. Higher propagule pressures increase the probability of invasion success^52^. In the high carrying capacity tanks, the bacterial density was, on average, 7.8× higher. Thus, these bacterial communities experienced a significantly lower propagule pressure when invaded. As marine larvae drink approximately 10^4^–10^6^ bacteria from the rearing water per day^10,53^, the larvae in the high carrying capacity water had a lower chance of being colonised or exposed to the invading bacteria. This observation might explain why higher survival and robustness are observed in high carrying capacity aquaculture systems such as recirculating aquaculture systems (RAS) and fed MMS systems^9,12,13,54^.

An unexpected observation was that the larvae from the FTS+ tanks were most robust to challenges but had the lowest survival at the end of the experiment. A likely explanation for this contrasting behaviour is that the larvae with low fitness already had died in the tanks. Consequently, when sampling from the tanks with few individuals left, the likelihood of selecting more robust larvae was higher in the FTS+ tanks. Thus, these results are likely biased towards a difference in population fitness means. The differences were caused by environmental conditions rather than a legacy effect.

Instead of a legacy effect from the initial rearing condition, microbial selection within the rearing tanks seemed to contribute to larval performance. Our rearing water investigations indicate that we were able to provide a continuous input of a stable microbiota from the MMS+ biofilter. Communities with high microbial community stability are usually found in K-selective environments^12,54^. Such environments typically have a stronger selection pressure and potentially a higher utilisation of available niches. In contrast, the three other treatments (MMS−, FTS− and FTS+) were prone to higher microbial turnover. Previous studies have documented that a large gap between the carrying capacity of the incoming and rearing water select for fast-growing opportunistic r-strategic bacteria^3^. Therefore, good rearing management should avoid r-selective environments, as most detrimental bacteria are r-strategists. We observed that excess available resources in the rearing tanks resulted in an enormous increase in bacterial density (132× in FTS+), indicating a strong r-selective environment. On the other hand, the MMS+ that received the same amount of resources, but added in the biofilter before the tanks, appeared to be K-selective, as the bacterial density was stable between the incoming and rearing water (1.2×). Thus, we obtained different selective environments in our treatments.

That the FTS+ rearing water was r-selected was also reflected by the ASVs linked to low survival. Although a difference in oxygen saturation might have contributed to mortality, 5 of the 52 ASVs linked to low survival had high 16S rRNA gene similarity to the type strain of *Moritella viscosa*. This bacterium is known to cause winter ulcers in cold-water fish such as Atlantic salmon and Atlantic cod^55^. Its increased abundance in the tanks with low survival might have contributed to the higher mortality in these tanks. For example, increased pathogen concentration has been shown to activate the adaptive immune systems^16^, making the fish more stressed. It should be noted that we did not study the larval microbiota. Vestrum et al. (2020) showed that an OTU belonging to the *Arcobacter* genus dominated the cod larval microbiota with abundances over 65%, whereas in the rearing water it never exceeded 2.3%^16^. Thus, investigations into the larval microbiome in this experiment might have allowed us to identify a potential pathogen with higher certainty.

The differences in survival between the treatments have two major implications. First, a protective legacy effect was not observed in the larvae. Instead, it seems like the microbial selection pressure in the rearing tanks provided a protective effect. The probability that a larva encounters a detrimental bacterium in K-selective environments is lower than in r-selected environments. This was apparent when comparing the high survival in tanks with the MMS+ treatment throughout the experiment with the very low survival in tanks that switched to FTS+. Thus, exposure to unfavourable conditions increases mortality regardless of how good the conditions are during the initial hatching. Secondly, when comparing the survival in the FTS+ and ‘FTS+ to MMS+’ tanks, there was no indication that improving the rearing environment led to higher survival. This indicates that if the larvae are exposed to unfavourable conditions early in life, their viability cannot be reversed by simply improving their environment. These observations have significant implications and illustrate that to obtain high survival and larval welfare it is vital to have good rearing conditions from the start and throughout the larval rearing period.

In conclusion, we found no evidence that the initial rearing condition left legacy effects on Atlantic cod larvae or their rearing water microbiota. Instead, the difference in carrying capacity between the intake and rearing water, the rearing water carrying capacity and the present water treatment had a much higher importance for larval viability and microbiota characteristics. We are the first to investigate legacy effects and report the lack of these effects during the first twenty days of marine Atlantic cod larvae rearing. Our study emphasises the importance of providing a beneficial rearing microbial environment throughout the rearing period to obtain high larval viability and bacterial water quality.

## Supplementary Information

Supplementary Information 1.

Supplementary Information 2.

Supplementary Information 3.

Supplementary Information 4.

Supplementary Information 5.

Supplementary Information 6.

## Data Availability

The sequencing data is available at ENA under study ERP138000 with accession numbers ERR9837055-ERR9837086. In addition, raw data for bacterial density, bacterial net growth potential, larval weight and larval survival (challenge and final) are available as supplementary files. All scripts used to perform data analysis and plot generation are available at https://github.com/madeleine-gundersen/legacy_effects_in_rearing_systems.
