# Supplementary material for: Aquaculture rearing systems induce no legacy effects in Atlantic cod larvae or their rearing water bacterial communities
Source: Sci Rep. 2022 Nov 17;12:19812. doi: 10.1038/s41598-022-24149-x (PMC9672056; doi:10.1038/s41598-022-24149-x)
Supplement: Supplementary file 1 — Supplementary Information 1. [file 41598_2022_24149_MOESM1_ESM.pdf]

Supplementary material for

Aquaculture rearing systems induce no legacy effects in Atlantic cod larvae or their rearing water bacterial communities

Authors: Madeleine S. Gundersen[1], Olav Vadstein[1] , Peter De Schryver[2, 3] and Kari Johanne Kihle Attramadal[1]

[1] Department of Biotechnology and Food Science, NTNU - Norwegian University of Science and Technology, Trondheim, Norway

[2] INVE Technologies NV, Dendermonde, Belgium

[3] Laboratory of Aquaculture and *Artemia* Reference Center, Ghent University, Ghent, Belgium

#Address correspondence to Madeleine Gundersen, madeleine.gundersen@ntnu.no

Supplementary tables and figures

Supplementary table 1: Rearing regime of Atlantic cod larvae stocked at a density of 100 larva/L in 100L tanks. From 3 DPH the larvae were fed three times a day with varying ratios of rotifers to artemia from 18 DPH.

<table>
<tr><td>DPH</td><td>0</td><td>1</td><td>2</td><td>3</td><td>4</td><td>5</td><td>6</td><td>7</td><td>8</td><td>9</td><td>10</td><td>11</td><td>12</td><td>13</td><td>14</td><td>15</td><td>16</td><td>17</td><td>18</td><td>19</td><td>20</td></tr>
<tr><td>Temperature [^oC]</td><td>9</td><td colspan="2">8</td><td colspan="2">9</td><td colspan="2">10</td><td colspan="2">11</td><td colspan="12">12</td></tr>
<tr><td>Light</td><td colspan="4">dark</td><td colspan="17">continuous light</td></tr>
<tr><td>WER [V/day^{-1}]*</td><td colspan="8">2</td><td colspan="13">4</td></tr>
<tr><td>Feed type</td><td colspan="3">none</td><td colspan="15">rotifers</td><td colspan="3">rotifers/artemia</td></tr>
<tr><td>n meals</td><td colspan="3">0</td><td colspan="15">3 (9am, 3pm, 9 pm)</td><td>3/1</td><td>2/2</td><td>1/3</td></tr>
</table>

* WER = Water exchange rate [tank volume day^{-1}]

Supplementary table 2: Number of flasks challenged on different sampling days. Tanks with FTS+ as initial rearing treatment were only challenged with invasion 8 DPH due to high mortality in the rearing tanks.

	**Challenge:**	**Invaded**	**Invaded**	**Transferred**	**Invaded**	**Transferred**
**Tank**	**Treatment**	**8 DPH**	**11 DPH**	**11 DPH**	**17 DPH**	**17 DPH**
1	MMS-	2	-	-	2	1
2	MMS-	2	2	1	2	1
3	MMS- to FTS-	2	2	1	2	3
4	MMS- to FTS-	-	2	1	2	1
5	MMS+ to FTS+	2	2	1	2	1
6	MMS+ to FTS+	2	2	1	2	1
7	MMS+	2	2	1	2	1
8	MMS+	-	-	-	2	1
9	FTS- to MMS-	2	2	1	2	1
10	FTS- to MMS-	2	2	1	2	1
11	FTS-	2	2	1	2	1
12	FTS-	-	-	-	2	1
13	FTS+ to MMS+	2	2	1	2	1
14	FTS+	2	-	-	-	-
15	FTS+	2	-	-	-	-
16	FTS+ to MMS+	-	-	-	-	-

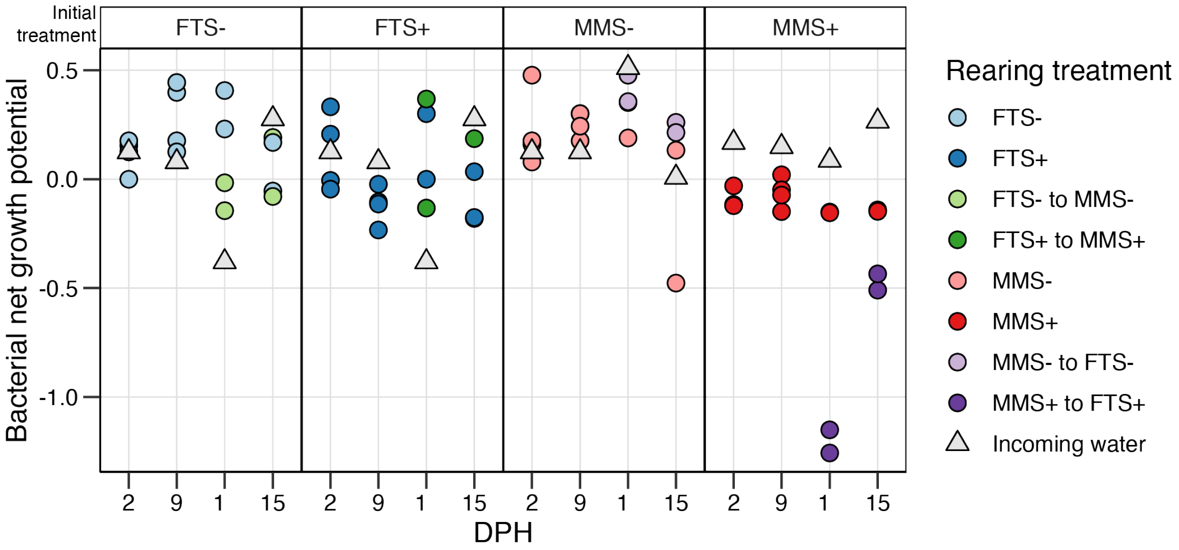

Supplementary figure 1: Bacterial net growth potential in incoming- and rearing tank water at various days post-hatching (DPH) presented as the log 10 bacterial density ratio between incubated and unincubated samples. Colours indicate rearing treatment, and shape signifies tank (●) and incoming water (▲).

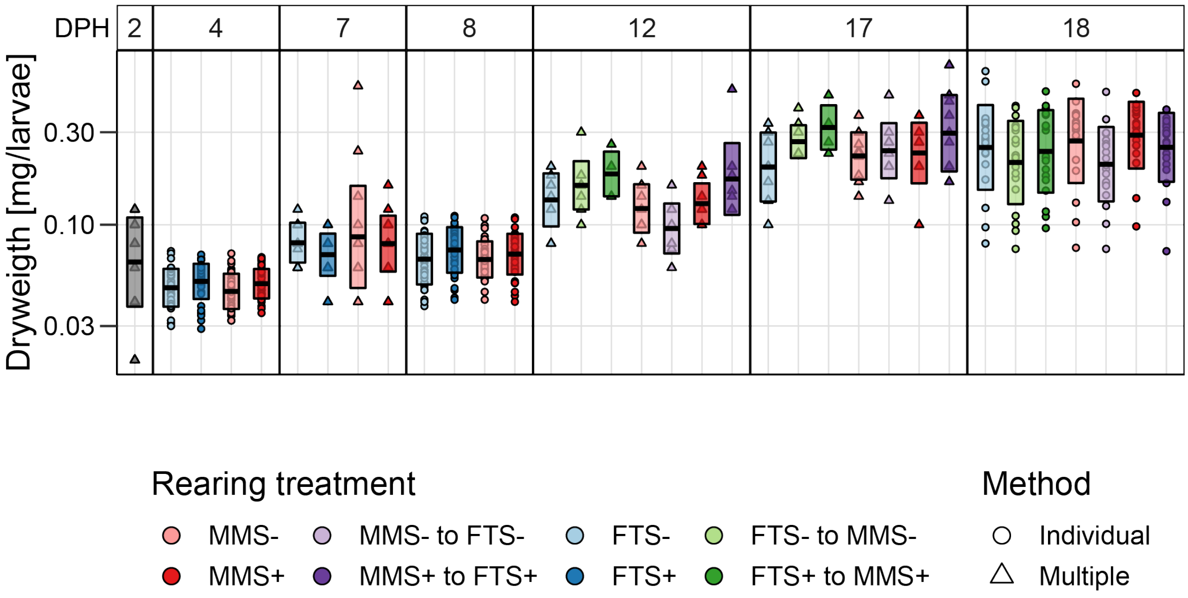

Supplementary figure 2: Log scaled dry weight [mg fish^-1] at various days post-hatching (DPH). Colours indicate the rearing treatment and filled or hollow indicates high or low carrying capacity. Boxplots represent mean dryweight±SD for each rearing treatment at each sampling day, with whiskers indicating maximum and minimum values recorded (n=15-25 and n=9-10 larvae/rearing tank for pooled (◆) and individual (●) weighing, respectively). At 2 DPH, n=10 larvae were chosen randomly and weighted to function as a baseline. Due to high mortality, no weight was measured for larvae reared in the FTS+ treatment from 12 DPH. On 12 and 17 DPH, only n=5 larvae were weighted for the FTS+/MMS+ treatment.

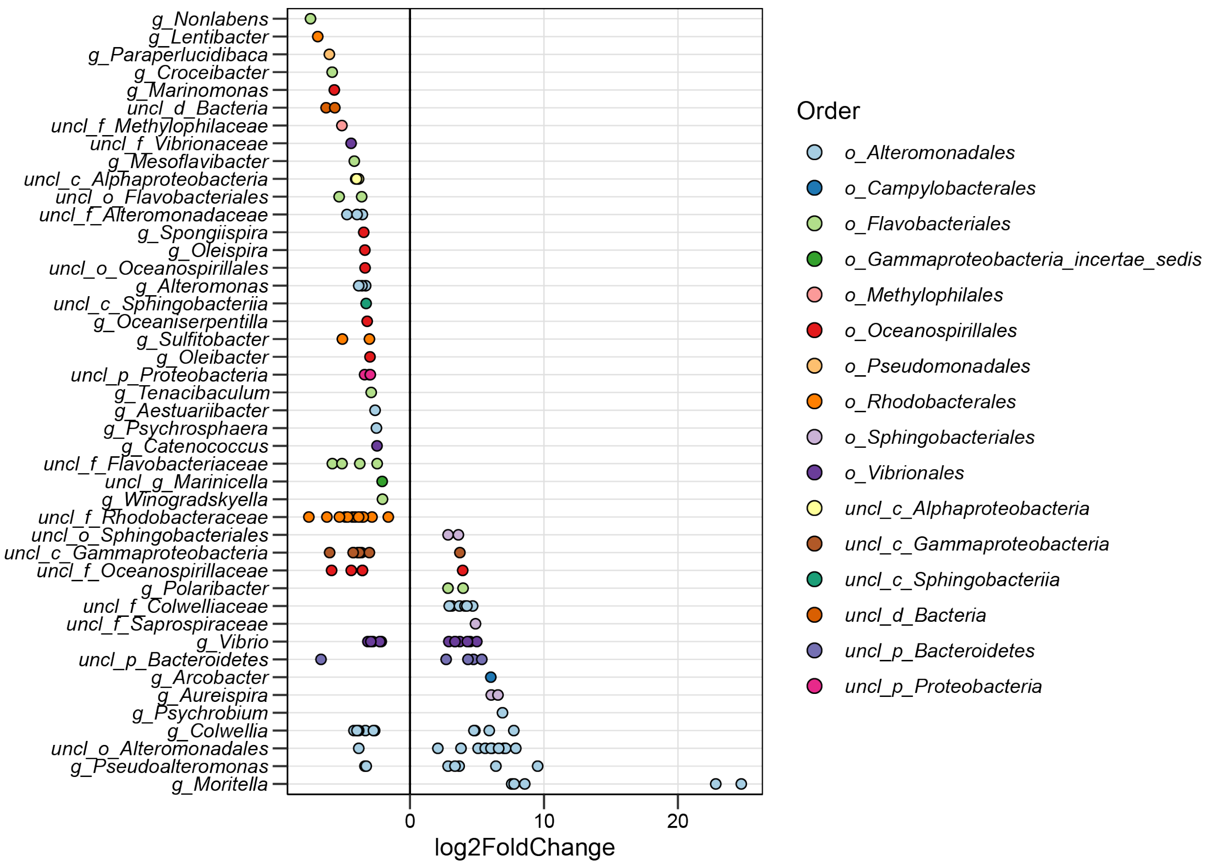

Supplementary figure 3: The log2 fold change in relative abundance for the ASVs in the rearing water with a significance level lower than 0.05 comparing samples with high vs low survival (FDR-adjusted DESeq2 p-values). Each ASV is represented by a point coloured according to its classification at the order level. The ASVs were grouped according to their lowest classification obtained along the y-axis (p = phylum, c = class, o = order, f = family, g = genus).

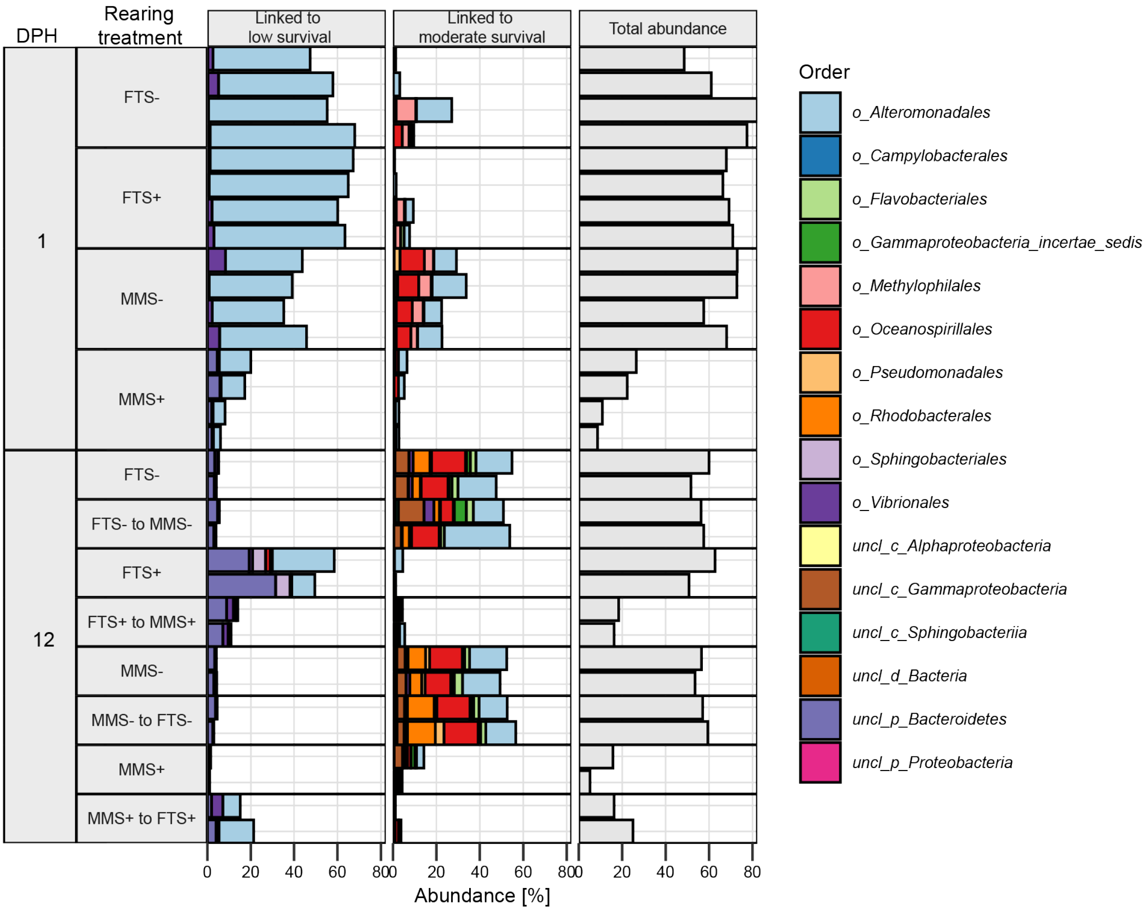

Supplementary figure 4: Abundances of the ASVs associated with high or low survival in each rearing water sample. These ASVs were identified through a DeSeq2 analysis and had an FDR-adjusted p-value < 0.05.

Supplementary table 3: RDP SeqMatch scores and matches for the five ASVs classified to the *Moritella* genus with a significant log2-fold change towards low survival. The RDP match score indicates the sequence similarity to the type strain (T) match.

ASV	Log2-fold change	RDP match score	RDP match
Zotu243	24.73	0.967	*Moritella abyssi* (T) ; 2693; AJ252022
		0.950	*Moritella viscosa* (T); NVI 88/478T; AJ132226
Zotu952	22.82	0.942	*Moritella viscosa* (T); NVI 88/478T; AJ132226
Zotu164	8.56	0.923	*Moritella viscosa* (T); NVI 88/478T; AJ132226
Zotu181	7.77	0.920	*Moritella viscosa* (T); NVI 88/478T; AJ132226
Zotu145	7.59	0.920	*Moritella viscosa* (T); NVI 88/478T; AJ132226

Quality assessment of the 16S rRNA gene amplicon sequencing data

The 16S rRNA gene amplicon dataset contained 450 369 sequence reads and 1315 ASVs, with a mean sequencing depth of 14 074 (±6418 SD) reads per sample. At the sequence depth cut-off of 3711 reads, the average slope of the rarefaction curve was 0.01, indicating that more ASVs could have been detected (**Supplementary figure 5**).

We evaluated the effect of ASV-table normalisation by investigating the Pearson correlation in α-diversity between the whole and normalised dataset. High correlation (R^2) and regression slopes close to one indicate that α-diversity is conserved in the normalized dataset. The diversity of order 1 in the normalised dataset correlated well with the full dataset ($R^2 = 0.99$) with a linear regression slope of 0.92. For richness, the slope was 0.49, and R^2 was 0.83 (**Supplementary figure 6**) and should thus be interpreted with caution.

Differences in community composition between the whole and normalised datasets were ordinated and projected with a symmetrical Procrustes analysis[1]. The analysis was performed using *procrustes*() from vegan. A low sum of squares indicates that the community composition patterns are conserved in the normalised dataset. Inspecting the differences in β-diversity community profiles between the whole and normalised dataset through a Procrustes analysis indicated that the community profiles were highly similar between the datasets (Sum of squares < 0.05 for abundance and incidence-based UniFrac and Bray-Curtis, **Supplementary figure 7**). This similarity indicates that the ASVs removed during normalisation had a minor impact on the β-diversity. In conclusion, the normalized ASV table was of high quality and satisfactorily represented the diversity, except for richness.

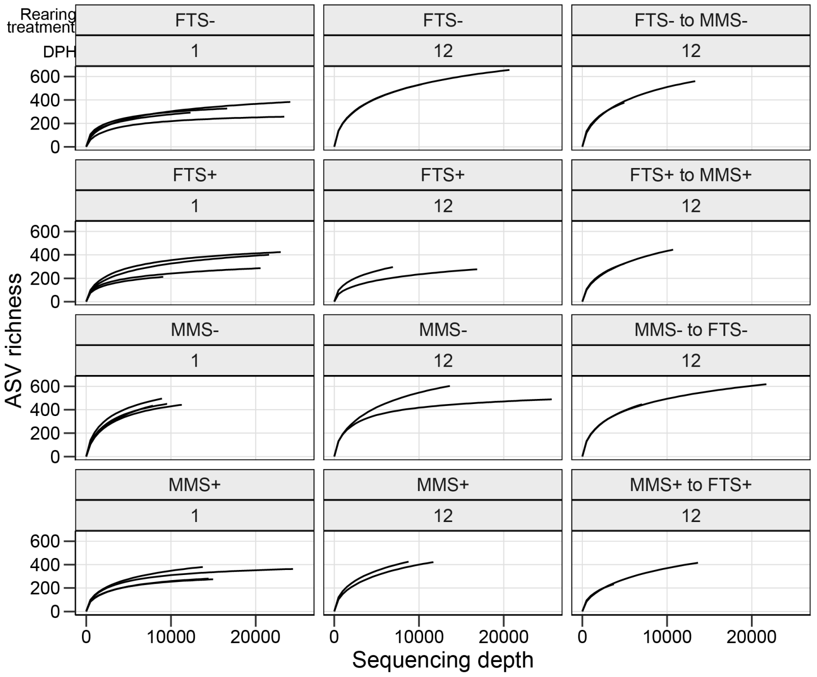

Supplementary figure 5: Rarefaction curve presenting the number of observed ASVs in the rearing water as a function of sequencing depth. One curve is estimated per sample. Each panel shows samples from the same rearing treatment sampled on the same day post-hatching (DPH).

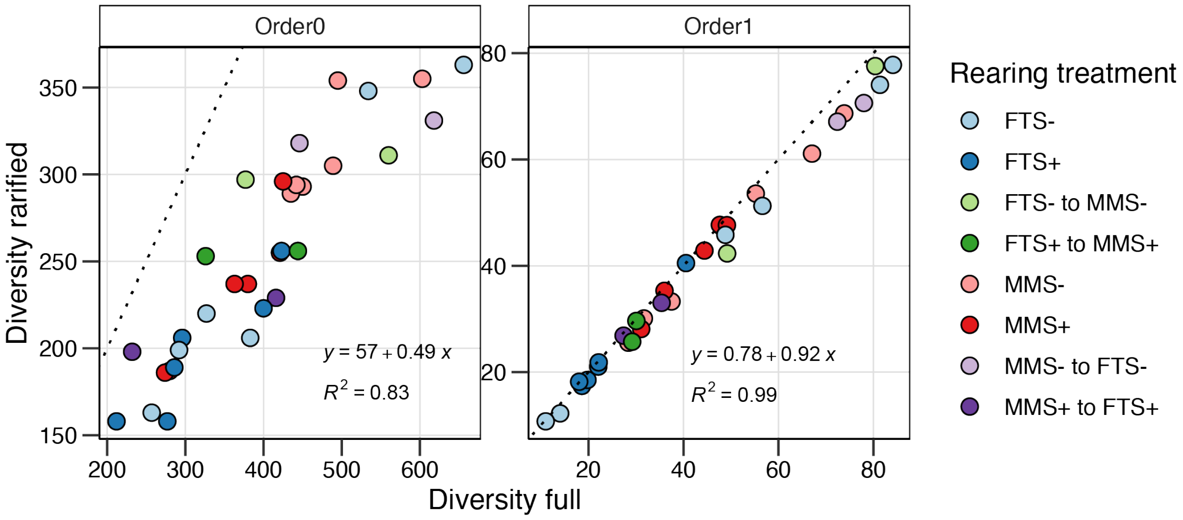

Supplementary figure 6: Relationship in α-diversity of order 0 (richness) and 1 between the normalised and full dataset. For normalisation, each rearing water sample was scaled to the lowest sampling depth and then rarefied. As a result, 25 ASVs were lost, and the normalised samples had 3711±0 (mean±SD) reads. Linear regression was used to obtain the equation and the sum-of-squares value. Colours represent the rearing treatment for each sample.

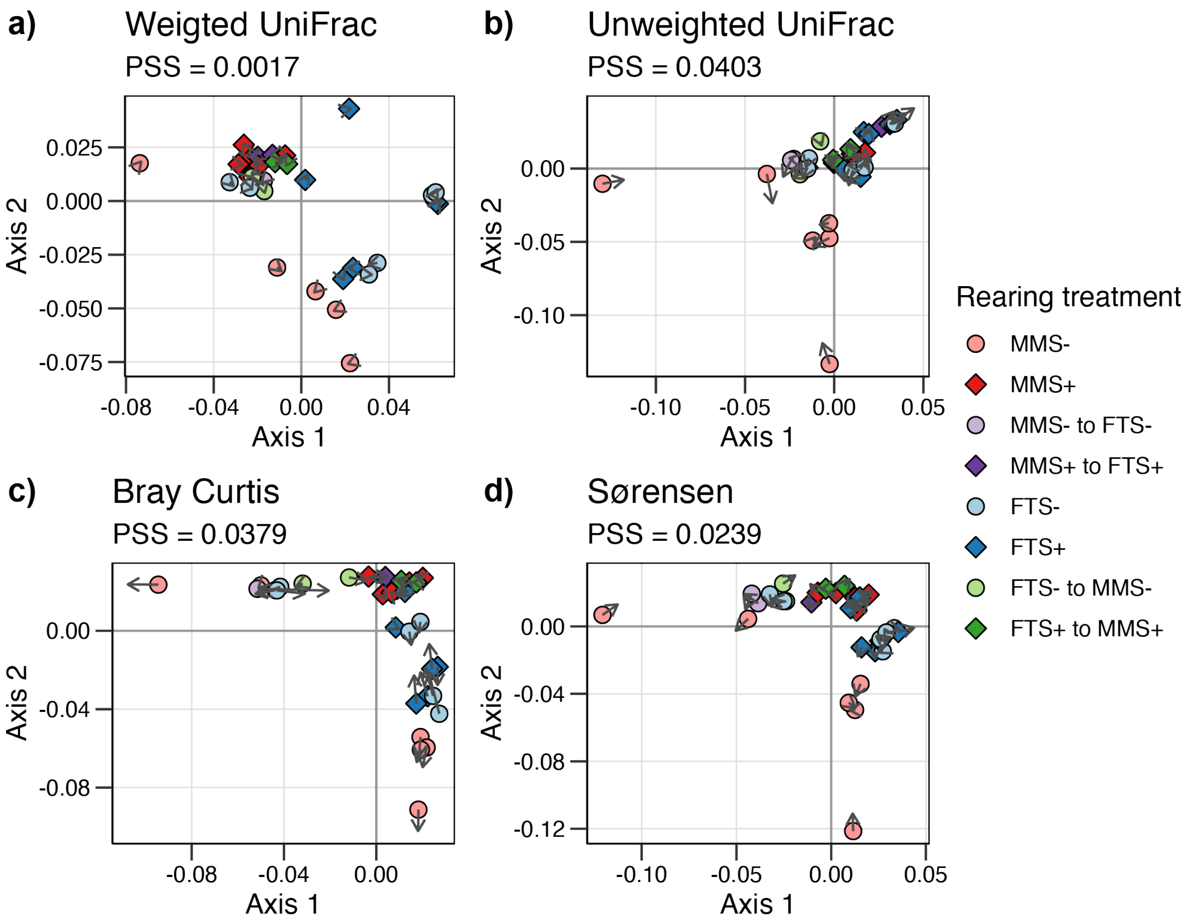

Supplementary figure 7: Procluster analysis indicating differences in rearing water community composition between the full and normalised dataset based on a) weighted UniFrac, b) unweighted UniFrac, c) Bray-Curtis, and d) Sørensen distance indices. PSS indicates the Procluster Sum of Squares.

References

1. Peres-Neto, P. R. & Jackson, D. A. How well do multivariate data sets match? The advantages of a Procrustean superimposition approach over the Mantel test. *Oecologia* **129**, 169–178 10.1007/s004420100720 (2001).
